# Second-Order Nonlinear Circular Dichroism in Square Lattice Array of Germanium Nanohelices

**DOI:** 10.1021/acsphotonics.4c00721

**Published:** 2024-08-16

**Authors:** Grégoire Saerens, Günter Ellrott, Olesia Pashina, Ilya Deriy, Vojislav Krstić, Mihail Petrov, Maria Chekhova, Rachel Grange

**Affiliations:** † ETH Zurich, Department of Physics, Institute for Quantum Electronics, Optical Nanomaterial Group, 8093 Zurich, Switzerland; ‡ Department of Physics, Friedrich-Alexander-Universität Erlangen-Nürnberg (FAU), 91058 Erlangen, Germany; ¶ School of Physics and Engineering, ITMO University, 191002 St. Petersburg, Russia; § University of Brescia, Department of Information Engineering, Via Branze 38, 25123, Brescia, Italy; ∥ Qingdao Innovation and Development Center, Harbin Engineering University, Sansha road 1777, Qingdao, 266000, Shandong China; ⊥ Max-Planck Institute for the Science of Light, 91058 Erlangen, Germany

**Keywords:** second-harmonic generation, centrosymmetric crystals, circular dichroism, germanium nanohelices, inversion symmetry

## Abstract

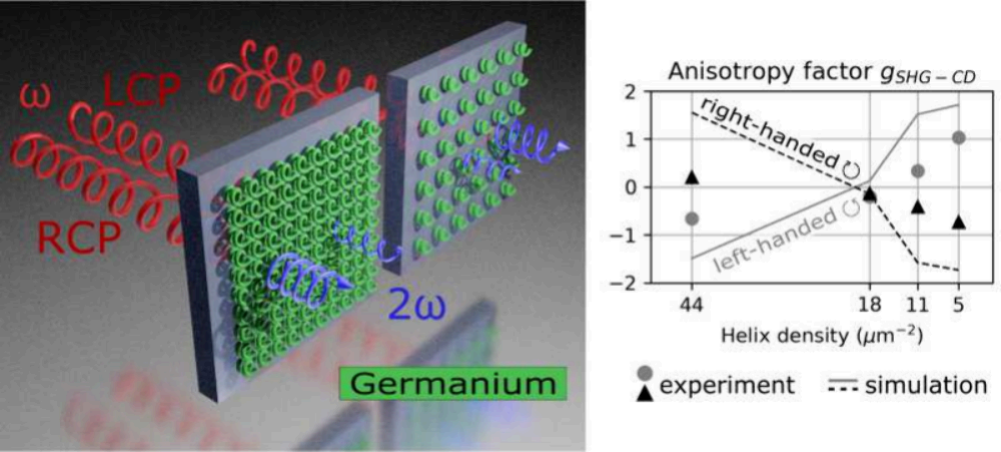

Second-harmonic generation (SHG) is prohibited in centrosymmetric crystals such as silicon or germanium due to the presence of inversion symmetry. However, the structuring of such materials makes it possible to break the inversion symmetry, thus achieving generation of second-harmonic. Moreover, various symmetry properties of the resulting structure, such as chirality, also influence the SHG. In this work, we investigate second-harmonic generation from an array of nanohelices made of germanium. The intensity of the second-harmonic displayed a remarkable enhancement of over 100 times compared to a nonstructured Ge thin film, revealing the influence of interaction between nanohelices. In particular, nonlinear circular dichroism, characterized through the SHG anisotropy factor *g*_SHG–CD_, changed its sign not only with the helix handedness but also with its density as well. We believe that our discoveries will open up new paths for the development of nonlinear photonics based on metamaterials and metasurfaces made of centrosymmetric materials.

## Introduction

To feature second-order nonlinear effects, a material needs to have no inversion symmetry. But even in materials without bulk second-order susceptibility χ^(2)^, the inversion symmetry can be lifted through structuring. The simplest example is breaking of an inversion symmetry at the surface of a planar structure. Second-order effects, such as second-harmonic generation (SHG), have been observed on the surfaces of metals and semiconductors already at the dawn of nonlinear optics.^1^ The surface contribution becomes significant even for noncentrosymmetric materials if their bulk response is suppressed by the orientation of the crystal sample^2^ or in nanoscale structures where surface-to-volume ratio is relatively high.^3^ In plasmonic (metal) nanostructures, SHG is considerably enhanced if the structuring breaks the inversion symmetry at subwavelength scales. Plasmonic metasurfaces with noncentrosymmetric, also 2D-chiral, meta-atoms can reach SHG efficiencies of η_SHG_ = *P*_SHG_/*P*_Fund_ = 10^–9^ at input powers *P*_Fund_ of few μW,^4^ where *P*_SHG_ is the measured SHG power. However, higher input powers are hardly tolerable because of the low damage threshold of plasmonic nanostructures.

Dielectric metamaterials have the advantage of higher damage thresholds. Besides, unlike in metallic structures, where the electric field is nonzero only on the surface, dielectric materials allow the nonlinear response from the bulk as well. The most efficient second-order nonlinear effects are observed in metasurfaces made of materials with high bulk second-order susceptibility χ^(2)^, such as GaAs, AlGaAs, GaP, LiNbO_3_ etc.^4−8^ Especially in the presence of geometric resonances, metasurfaces made of such materials enable SHG efficiency of up to 10^–5^ for pulsed pump^9^ or up to 10^–7^ for CW pump.^10^

Silicon (Si) and germanium (Ge), which are commonly used centrosymmetric all-dielectric materials, require techniques for second-order nonlinear applications, either enhancing the electric field at the surface^11−13^ or inducing externally an asymmetry in the crystal.^14−16^ However, these techniques are often limited to a specific material or a narrow bandwidth of operation wavelengths. For instance, a metasurface with asymmetric (T-shaped) silicon meta-atoms generated second-harmonic with an efficiency of 10^–9^ under pumping with 3 mW.^17^ However, this effect was attributed solely to a resonance with a high (10^5^) quality factor.

**Figure 1:**
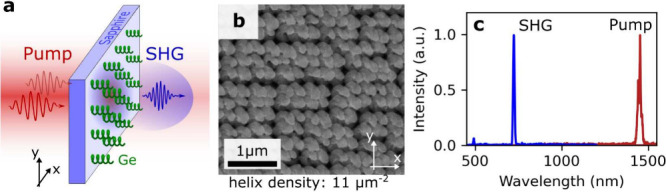
(a) Schematic of Ge nanohelices arranged in a square lattice on a transparent sapphire substrate and generating second-harmonic light in the visible. (b) Top scanning electron microscopy (SEM) image of closely packed Ge nanohelices with a density of 11 nanohelices per μm^2^. (c) Spectra of the pulsed laser light at 1450 nm wavelength (Pump) and of the filtered signal (SHG). The filtered intensity peak is exactly at half the wavelength of the pump, indicating SHG from the Ge nanohelices.

In this paper, we structured a high-index semiconductor material into three-dimensional (3D) chiral elements,^18^ breaking the central symmetry not on the atomic level, as is typical, but on the subwavelength level. We investigated SHG from Ge nanohelices, as shown schematically in Figure 1a, and demonstrated that the helical structure can yield a second-order nonlinear response exceeding the surface contribution by more than 1 order of magnitude. To support our experimental findings, we conducted simulations using the finite element method (FEM), through the full-wave electromagnetic modeling software COMSOL Multiphysics, comparing in particular SHG from nanohelices and from centrosymmetric tori.

While circular dichroism is expected from such chiral structures and has already been studied in the linear optical regime,^19^ its manifestation in the SHG enables different applications - for instance, in nonlinear chiral sensing and imaging for material science and biology.^20−23^ Circular dichroism of the SHG is quantified through the SHG anisotropy factor *g*_SHG–CD_, in line with established methodologies:^24^
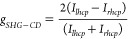
1where *I*_l/rhcp_ is the intensity of the left- and right-handed circular polarized light, respectively. Centrosymmetric structures, such as Cu or Ge halides,^24,25^ plasmonic nanoparticles,^26,27^ dielectric dimers,^28^ perovskites^29^ or dielectric and plasmonic metasurfaces^30−32^ have demonstrated circular dichroism of the nonlinear response in the visible and near-infrared region. Recently, there has also been a significant progress in the theoretical description of the SH dichroism in nanoantenna and metasurface structures.^33,34^ Here we measure, for the first time, the SHG anisotropy in pure Ge, with a factor *g*_SHG–CD_ that takes values up to 1 and changes sign around a density of 18 nanohelices per μm^2^. This experimental observation is further supported by our numerical simulations, which predict a maximal value of *g*_SHG–CD_ = 1.7 and the change of sign for similar nanohelix densities. We conclude by discussing the evidence of a collective effect, which was already observed in the linear optical regime.^19^

## Results and Discussion

We fabricated square-array arrangements of Ge nanohelices with subwavelength period and helical pitch, using the glancing angle deposition (GLAD) procedure as explained in our previous work.^19^ A top scanning electron microscopy image (SEM) is given in Figure 1b for the sample with a density of 11 nanohelices per μm^2^. Here, we defined the two lattice axes of the square array as x- and *y*-axis. The fabrication method as well as additional images can be found in Section S1 of the Supporting Information To investigate the impact of collective effects on the SHG, samples were grown with four different nanohelix densities, ranging from 44 to 5 helices per μm^2^, corresponding to a spacing of 150 and 450 nm, respectively. For all the samples, the helical diameter is *D* = 190 nm and the helical pitch is *p*_h_ = 360 nm, with 2 turns, resulting in a total height of 720 nm (see also Figure S3 in the Supporting Information).

We performed the nonlinear measurements using a near-infrared tunable femtosecond laser as the excitation light source. The laser’s power and polarization were controlled by motorized rotating half-waveplate retarders before and after a polarizing beam splitter, respectively. Switching between linearly- and circularly polarized light could be achieved by adding and rotating a quarter-waveplate. The beam was then focused onto the center of an array (100 × 100 μm^2^), resulting in a spot diameter of approximately 25 μm. The signal was collected using a 20x objective, filtered through two high-pass filters, and captured by either a CMOS camera or a spectrometer. Figure 1c shows the spectra of the pump and the filtered signal, confirming the detection of just the SHG, namely at exactly half the excitation wavelength. Additional details regarding the setup and the procedure for the SHG power measurement can be found in Section S2 of the Supporting Information, together with further characterization of the SHG for different wavelengths and powers.

**Figure 2:**
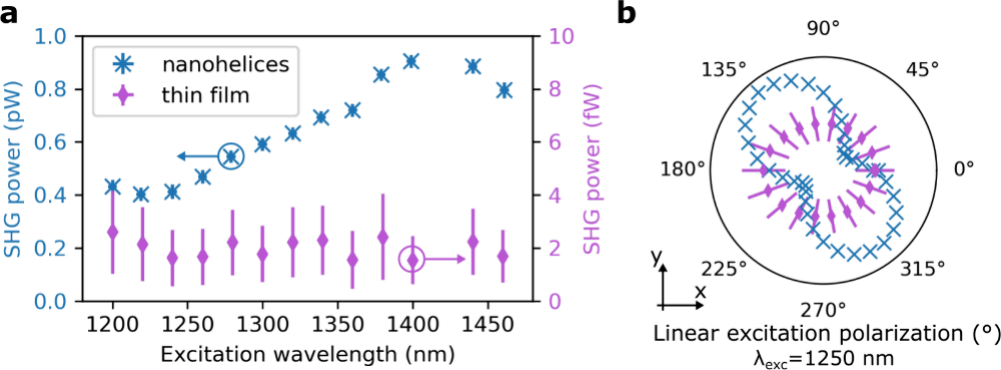
(a) SHG power measured at different excitation wavelengths from closely packed Ge nanohelices (blue, 44 nanohelices per μm^2^) and a comparison 50 nm thin Ge film (purple). The SHG intensity from the nanohelices is more than 100 times higher than that of a thin film. (b) Normalized SHG intensity for different excitation polarizations from the same nanohelix array as in (a) (blue crosses) and the Ge thin film (purple diamonds) at an excitation wavelength of 1250 nm.

The SHG emission from Ge nanohelices under an excitation power of 100 mW was observed in transmission using the integration time of a few seconds. For further comparison, we measured the SHG intensity of a 50 nm thin film of Ge, as shown in Figure 2a. The thin film emitted a barely visible SHG signal, while closely packed Ge nanohelices demonstrated a substantial 100-fold SHG increase over the whole excitation range, from 1200 to 1400 nm. Meanwhile, we calculated the surface increase from a thin film to Ge nanohelices (see Section S3 of the Supporting Information) and obtained only a factor of 12 for closely packed Ge nanohelices compared to a bare thin film. Similarly, we measured the SHG intensity for different densities of nanohelices and calculated the corresponding surface increase. In all cases, the surface increase of the nanohelix compared to a thin film was 1 order of magnitude smaller than the SHG increase. This lower increase indicates that SHG originates primarily from the central symmetry breaking through the subwavelength structuring, and not from the surface. To further differentiate the effect of the surface or structured SHG, we developed a simulation model to compute the SHG from an array of nanostructures. Our model is described in Section S4 and S5 of the Supporting Information and takes into account surface dipolar and bulk quadrupolar contributions to the second-order polarization in centrosymmetric materials.^11,35^ We conducted simulations of the Ge nanohelix array and compared it with a torus array, which is centrosymmetric and similar in volume, as well as with a thin film. The simulation results, given in Section S6 of the Supporting Information, indicate no circular dichroism from the torus array. Furthermore, the SHG intensity from a torus array is similar to the one for a planar structure. These calculations supported the experimental results, namely that the subwavelength structuring is the origin of the circular dichroism of the SHG and that the second-order nonlinear signal emerges from the noncentrosymmetry of the nanohelices.

Even though the bandgap of Ge is around 1900 nm, SHG could still be observed in the near-infrared. The absorption coefficient at the excitation (SHG) wavelength of 1400 nm (700 nm) is around 1.1 μm^–1^ (7.5 μm^–1^) leading to 93% (40%) of the light still transmitted through the 120 nm thick Ge, which is equivalent to two turns of the nanohelix.

The SHG intensity was also measured for different excitation polarizations. The polar plots, which were obtained by rotating the second half-wave plate (without the quarter-wave plate added), reveal that nanohelices emitted stronger SHG for specific polarizations, while the Ge thin film produced an SHG signal independent of the excitation polarization, as shown in Figure 2b. The origin of the polarization dependence of the nanohelix could be found in the start and end point of the nanostructure, which restrained the possible material polarization directions. The calculated electric field at these points also showed higher intensities, as shown in the Section S7 of the Supporting Information

**Figure 3:**
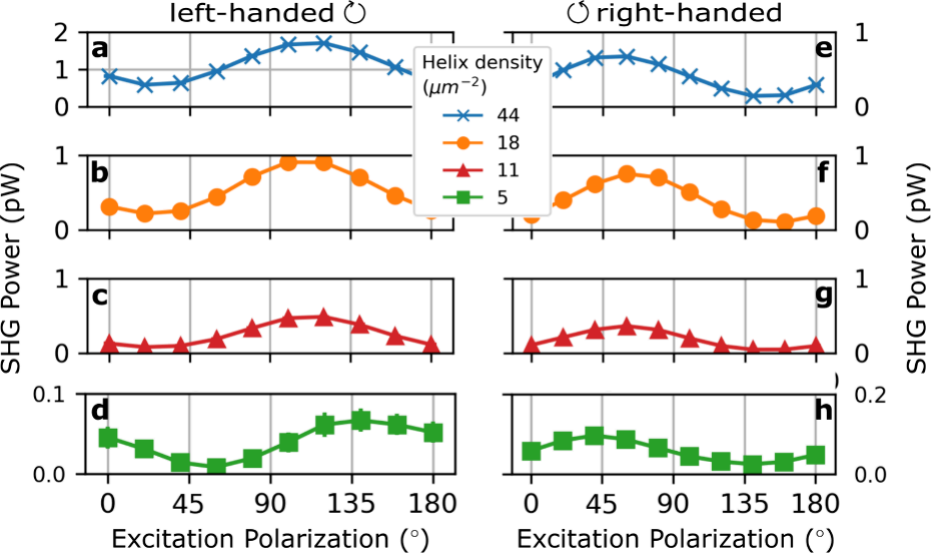
Linear polarization orientation dependence of the SHG power from (a)-(d) left-handed and (e)-(h) right-handed Ge nanohelices at an excitation wavelength of 1450 nm. Results are shown from the highest to the lowest density of helices (top to bottom). The highest SHG efficiency is observed for a polarization around 120° for left-handed nanohelices, relatively to the horizontal *x*-axis (see Figure 1), and around 60° for right-handed nanohelices.

We investigated further the linear polarization dependence of the SHG from Ge nanohelices with various densities and opposite handedness, as shown in Figure 3. The SHG power was measured at a wavelength of 1450 nm, where the maximum signal was observed, and at an average power of 100 mW. Even for lower nanohelix densities, the SHG possessed a strong dependence on the orientation of the linear polarization, peaking at around 120° (60°) for left-handed (right-handed) nanohelices relatively to the horizontal *x*-axis (see Figure 1). This is similar to Figure 2b. The measured strong polarization dependence and the impact of the handedness were also observed in the simulations, as shown in Section S8 of the Supporting Information

We defined the SHG anisotropy as ρ_SHG_ = (*I*_max_ – *I*_min_)/(*I*_max_ + *I*_min_), with *I*_max_ and *I*_min_ being the maximum and minimum SHG intensities, respectively, measured for two different linear polarizations. In Table 1, we give the SHG anisotropy ρ_SHG_, which ranges from 0.5 to 0.6 for closely packed nanohelices to 0.7–0.8 for a density of 11 nanohelices per μm^2^. These values of SHG anisotropy are smaller than the ones obtained numerically, as shown in the Section S8 of the Supporting Information This lower value could be related to the quality of the nanohelix, as defects or imperfections could reduce the polarization dependence.

**Table 1:** Measured SHG Anisotropy (ρ_SHG_) from Left-Handed and Right-Handed Ge Nanohelice Samples with Linearly Polarized Pump Light

	ρ_SHG_
Helix density (μm^–2^)	left-handed helices | right-handed helices
44	0.49 | 0.65
18	0.61 | 0.75
11	0.71 | 0.77
5	0.77 | 0.60

**Figure 4:**
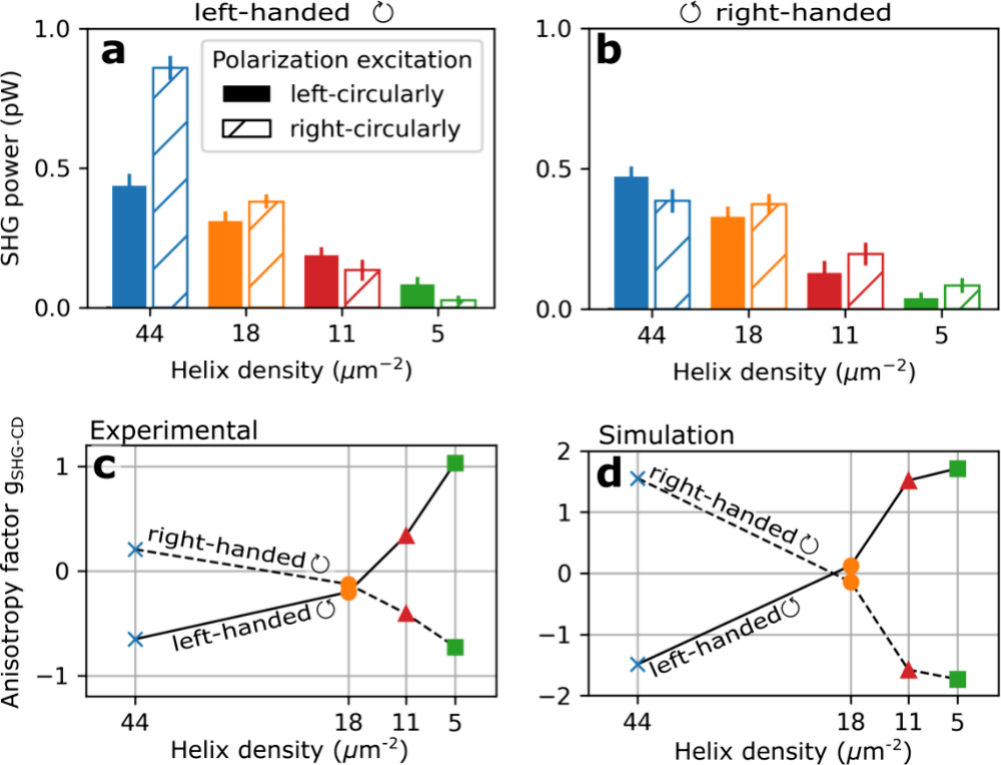
SHG power from (a) left-handed and (b) right-handed Ge nanohelices arranged from the highest to the lowest density of nanostructures, progressing from left to right. Strong SHG efficiency is observed from closely packed left-handed helices under right-circularly polarized light and from closely packed right-handed helices under left-circularly polarized light. This nonlinear circular dichroism reverses with low-density nanohelices. (c,d) SHG anisotropy factor *g*_SHG–CD_ measured (c) and simulated (d) for different densities and handednesses of the nanohelices.

Similarly as in our previous study in the linear optical regime,^19^ we measured the circular dichroism of the SHG at 1450 nm excitation wavelength and 100 mW average power. We observed a strong nonlinear circular dichroism that switches with the nanohelix’s handedness. The SHG from closely packed left-handed nanohelices was stronger for right-circularly polarized light (see Figure 4a), while from right-handed nanohelices, it was stronger for left-circularly polarized light (see Figure 4b). However, this circular polarization dependence reversed with an increase in the period. The SHG power from loosely packed left-handed nanohelices favored left-circularly polarized light, and conversely from loosely packed right-handed nanohelices.

The SHG anisotropy factor *g*_SHG–CD_ is illustrated in Figure 4c with different helix densities in the horizontal axis. Interestingly, we observed a zero crossing between 18 and 11 helices per μm^2^. The values of *g*_SHG–CD_ varied from 0.20 to −0.72 for right-handed nanohelices and from −0.65 to 1.0 for left-handed nanohelices. In comparison, the calculated *g*_SHG–CD_ varied from ±1.5 for the highest density of helices to ∓1.7 for the lowest, as shown in Figure 4d. A change of signs for nanohelix densities around 18 μm^2^ was also visible. We suggest that this change is due to destructive interference arising at certain periods, similarly to the change in circular dichroism that can be observed in plasmonic nanostructures when changing the density.^36,37^ The discrepancy between the experimental and simulated results, especially for left- and right-handedness, could again be explained by the sample quality. The comparison of SHG from different samples grown with similar parameters is shown in Section S9 of the Supporting Information From this, it is possible to estimate the uncertainty in the SHG measurement arising from fabrication. Nevertheless, simulations revealed a resonance close to the second-harmonic wavelength, which, with increasing nanohelix density, shifted toward the longer wavelength region of the spectrum. This is shown in Section S7 of the Supporting Information Our calculations showed also that increasing the helical diameter also leads to a similar change of sign in the circular dichroism, as shown in section S10 of the Supporting Information

**Figure 5:**
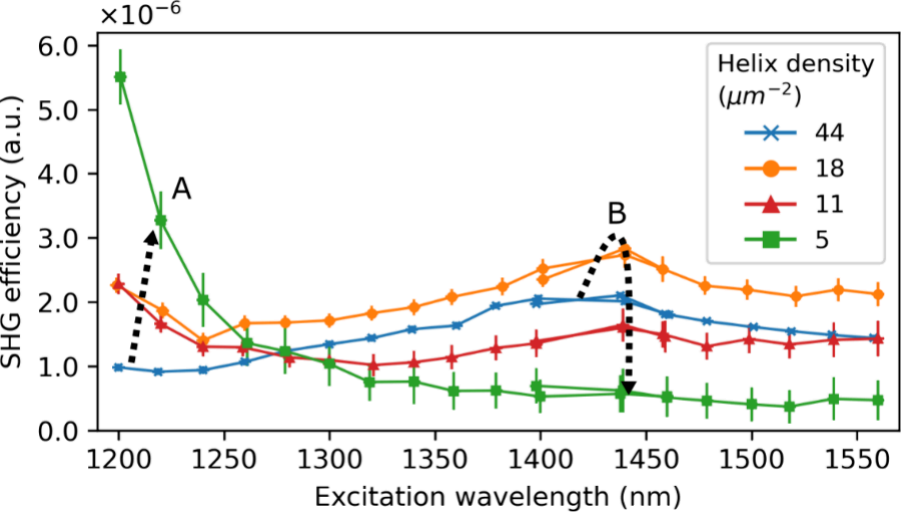
SHG intensity spectrum from Ge nanohelices with different densities. Plotted as the SHG power normalized to the excitation power and the nanohelix density. For higher densities, the strongest SHG is observed around 1450 nm excitation, while for lower densities, the SHG intensity increases more drastically at shorter wavelengths and with decreasing density. Two resonances are observed (A,B) which appear for different densities.

To further investigate the collective effect leading to this destructive interference of the SHG, we measured the SHG intensity for different excitation wavelengths and normalized them to the nanohelix density, as shown in Figure 5. We observed that the SHG intensity per nanohelix density increased by a factor of 5 with larger spacing between nanohelices and with an excitation wavelength around 1200 nm (resonance A in Figure 5). We interpret this difference as an indication of a collective effect, where emission from densely packed Ge nanohelices interferes destructively. While the efficiency per nanohelix increases with decreasing nanostructure density, further exploration is needed - for example, by testing samples with different helical pitches or periods, as well as measuring SHG below 1200 nm. A maximum SHG intensity per nanohelix density is observed for a density of 18 per μm^2^. This second resonance (B) may arise from constructive interference at a specific period (see also comparison in Section S11 of the Supporting Information). Note that the measurement was done in two steps, between 1200 and 1460 nm, and between 1400 and 1560 nm as it required a modified laser configuration at longer wavelengths (the pump laser wavelength was set to 820 nm instead of 800 nm). We added a correction factor of 1.45 for the second range, which took into account the new optical properties of the excitation laser, for instance the different pulse durations. We also observed that the SHG dependence on polarization diminishes for all Ge nanohelices when exciting with shorter wavelength, as detailed in Section S12 of the Supporting Information

These insights hold potential for developing efficient arrays of nonlinear emitters from a wide range of materials, not being limited by the bulk χ^(2)^ tensor. We develop a model of an ultrathin film with an effective second-order nonlinear tensor to predict SHG intensity and circular dichroism. Additionally, we calculate the conditions for achieving maximum circular dichroism based on the effective nonlinear tensor. For detailed information, please refer to sections S13–S15 in the Supporting Information Possible designs based on rescaling the structure should also give control over a broad range of excitation wavelengths.

## Conclusion

In summary, we fabricated periodic nanohelices arrays from Ge, a centrosymmetric material, and successfully measured SHG from it. The measured intensity was greatly enhanced compared to a Ge thin film, by a factor of more than 100. This two-order of magnitude enhancement could not be explained by the increase of surface only, which would increase only by about 12 times for the closest packed helices. We suggested a collective effect, further supported by our findings using different linearly and especially circularly polarized light. We measured the circular dichroism with the SHG anisotropy factor and obtained values of up to 1 depending on the helix density. This SHG anisotropy factor changed sign for helices grown with different handedness, as expected, but also changed sign with nanohelix densities around 15 μm^–2^. Finally, we measured the SHG spectrum from 1200 to 1560 nm and compared the SHG efficiency normalized to the nanohelix density. We observed at shorter wavelengths a much higher nonlinear signal for lower nanohelix densities, suggesting the role of collective effects in shaping their optical response. Our results present new opportunities for second-order nonlinear optical devices with strong optical activity for applications in all-optical signal processing, spectroscopy or nonlinear optics.

## Supporting Information Available

Additional SEM images of the sample, details on the setup for nonlinear characterization, calculations of the surface increase with nanohelices compared with a thin film, model for SHG calculations in centro-symmetric nanostructures, numerical simulations of the spectrum, influence of chirality on nonlinear emission, calculation and measurement of the SHG intensity dependence on the incoming laser’s polarization, measurement on SHG intensity uncertainty, and finally, measurement of polarization dependence with excitation wavelength (ZIP)
