# Supplementary figures and images for: Second-Order Nonlinear Circular Dichroism in Square Lattice Array of Germanium Nanohelices
Source: ACS Photonics. 2024 Aug 16;11(9):3630–5. doi: 10.1021/acsphotonics.4c00721 (PMC11413925; doi:10.1021/acsphotonics.4c00721)

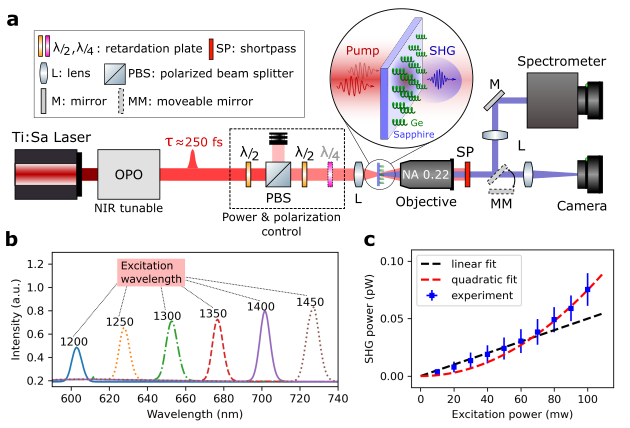

Supplement: Supplementary file 1 — ph4c00721_si_001.zip [file ph4c00721_si_001.zip › Fig_Suppl_Setup.png]

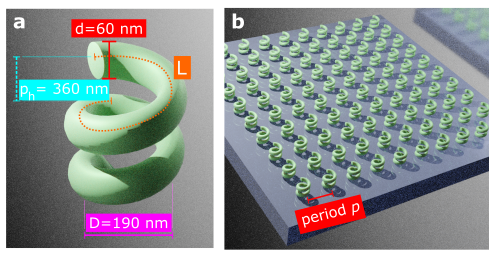

Supplement: Supplementary file 1 — ph4c00721_si_001.zip [file ph4c00721_si_001.zip › Fig_Suppl_Dim.PNG]

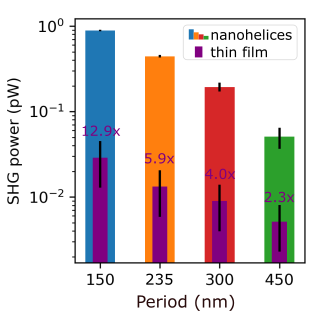

Supplement: Supplementary file 1 — ph4c00721_si_001.zip [file ph4c00721_si_001.zip › Fig_Suppl_Comp_Film.PNG]

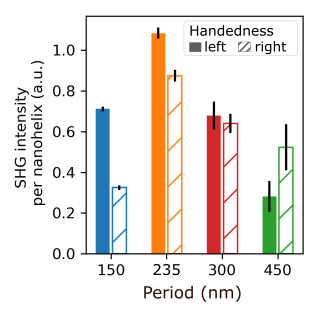

Supplement: Supplementary file 1 — ph4c00721_si_001.zip [file ph4c00721_si_001.zip › Fig_Suppl_SHG_perHelix_1450nm.PNG]

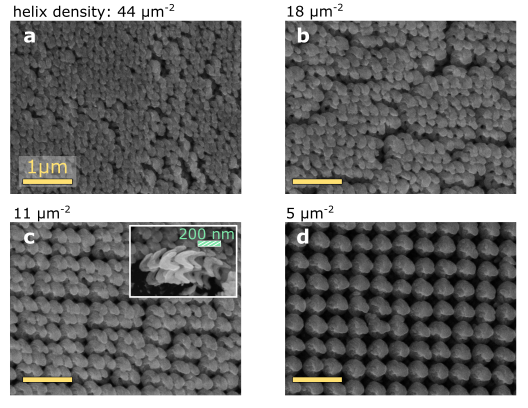

Supplement: Supplementary file 1 — ph4c00721_si_001.zip [file ph4c00721_si_001.zip › Fig_Suppl_SEM.PNG]

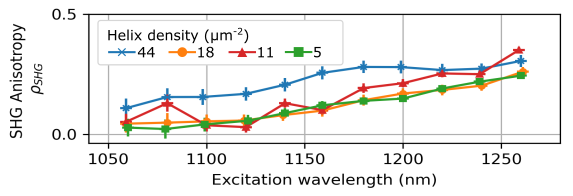

Supplement: Supplementary file 1 — ph4c00721_si_001.zip [file ph4c00721_si_001.zip › Fig_Suppl_Anisotopy.PNG]

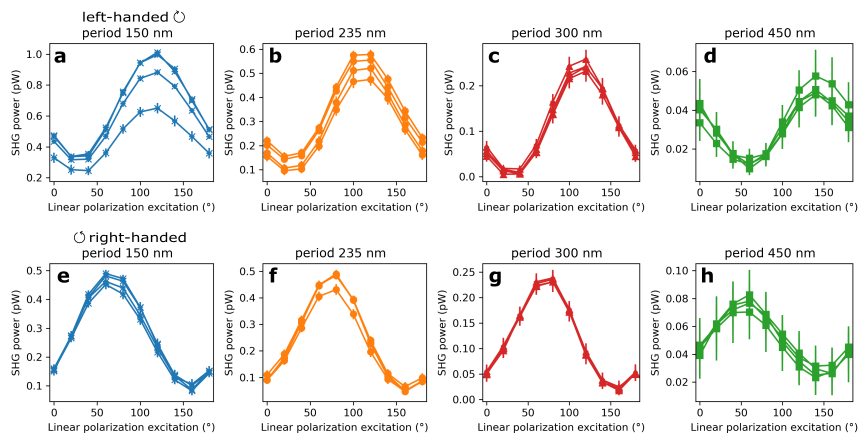

Supplement: Supplementary file 1 — ph4c00721_si_001.zip [file ph4c00721_si_001.zip › Fig_Suppl_Uncertainty.PNG]

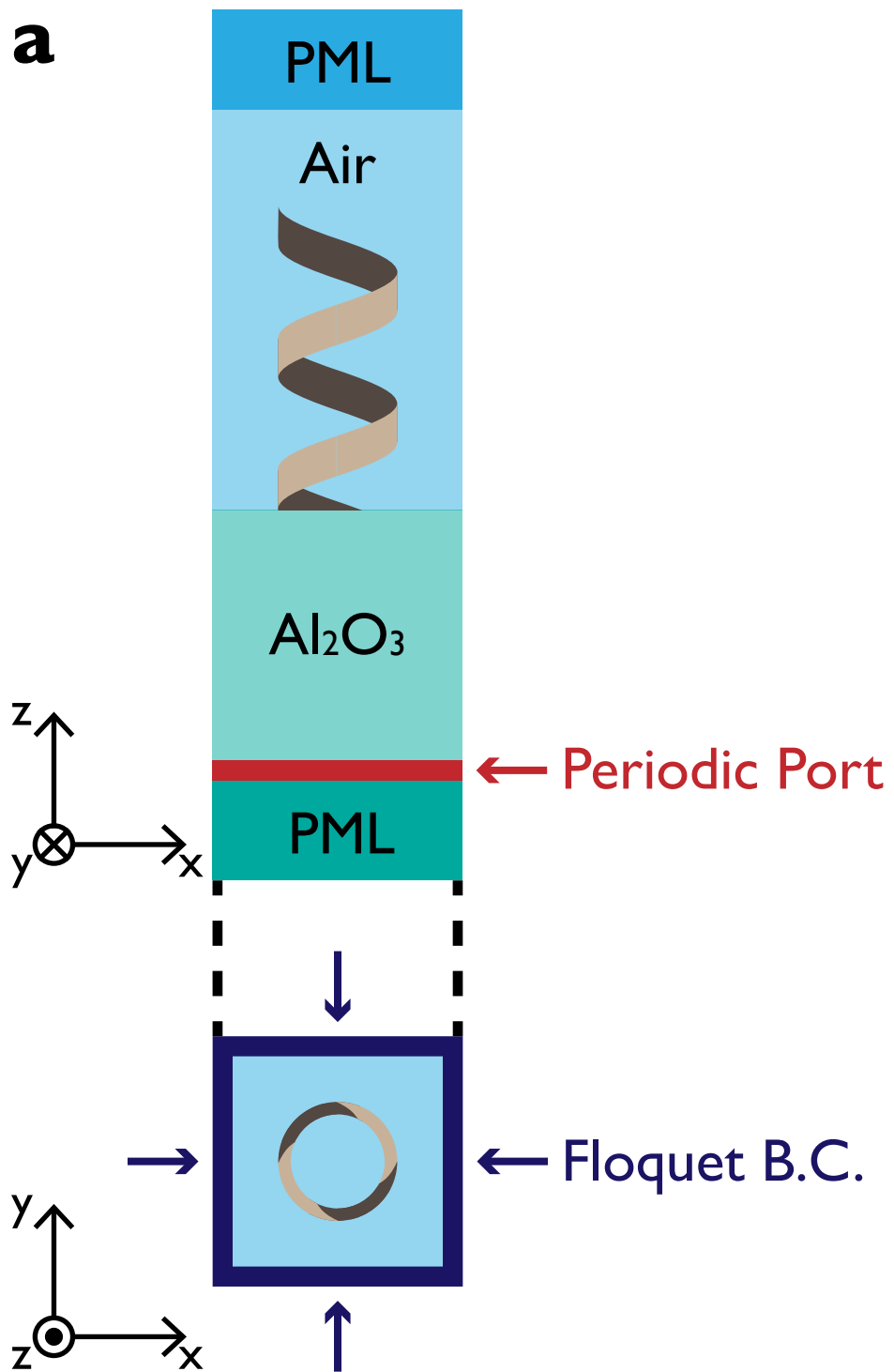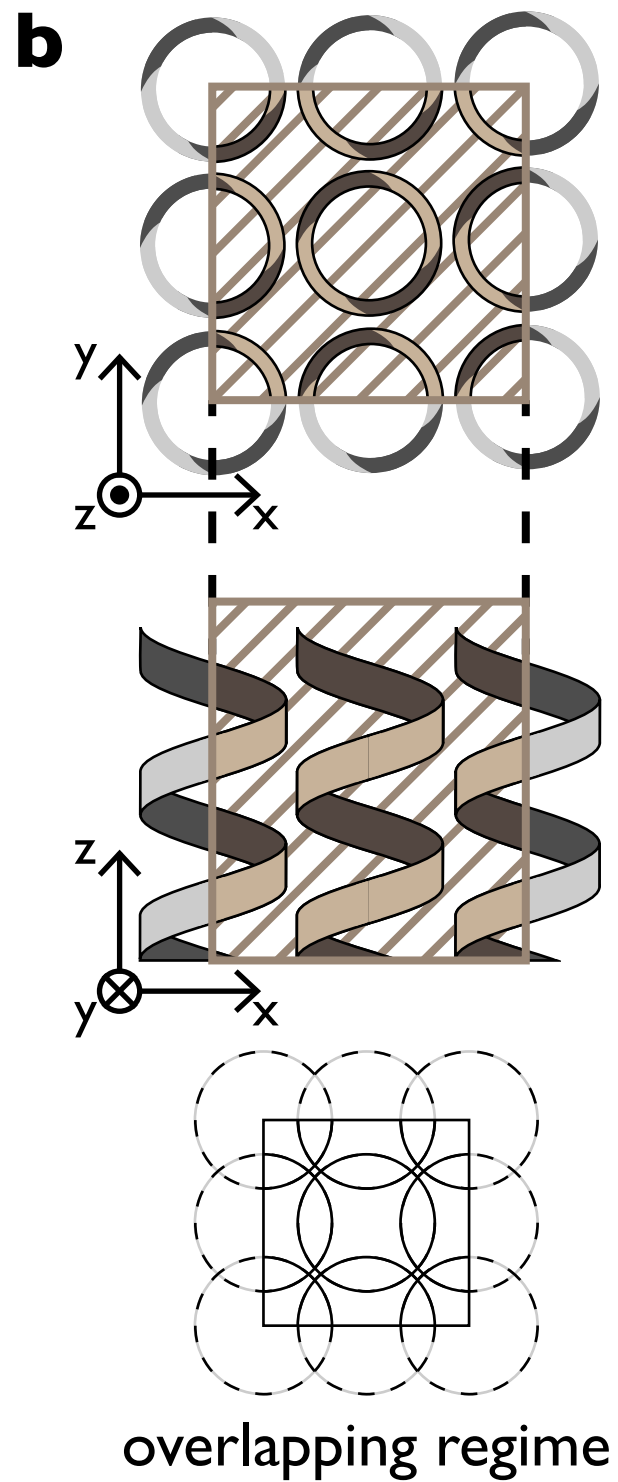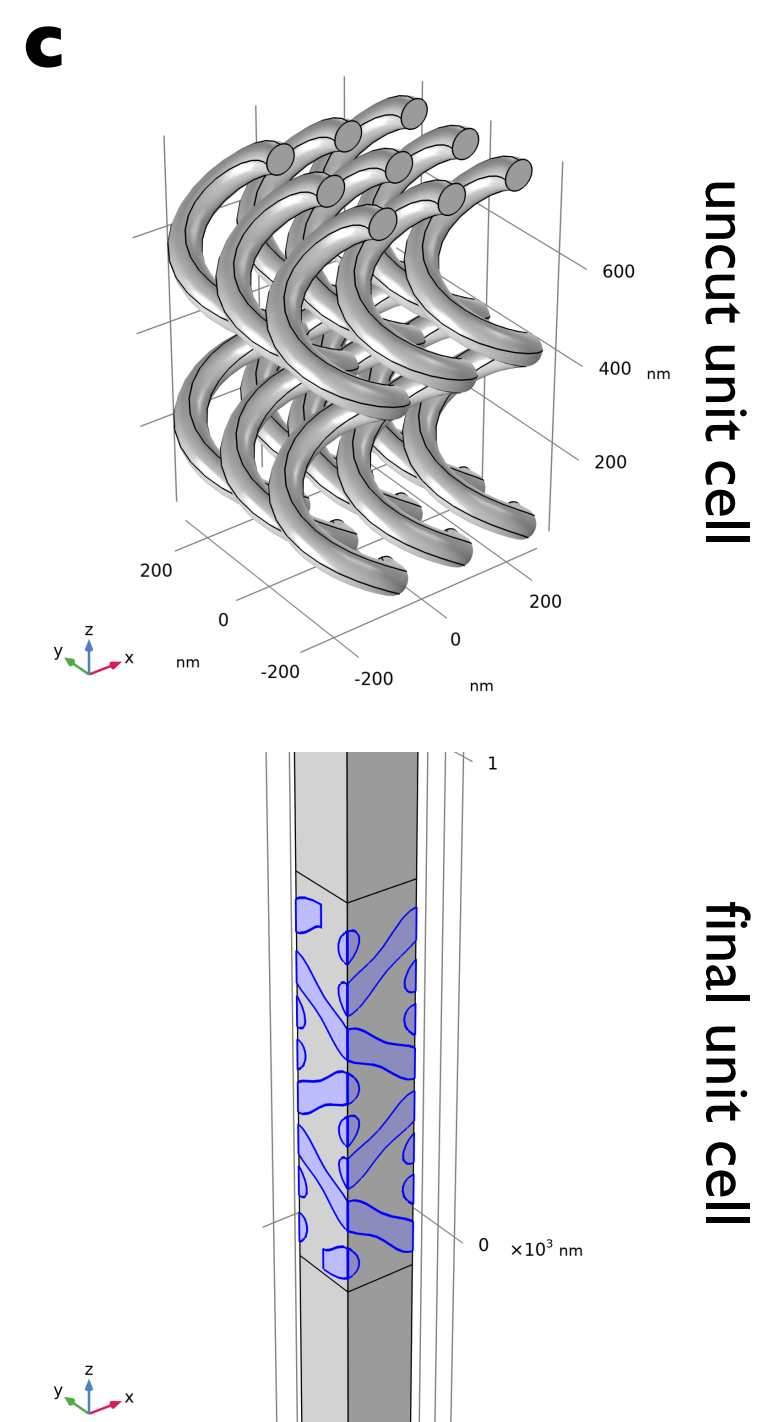

Supplement: Supplementary file 1 — ph4c00721_si_001.zip [file ph4c00721_si_001.zip › Fig_Suppl_numerical_geometry.pdf]

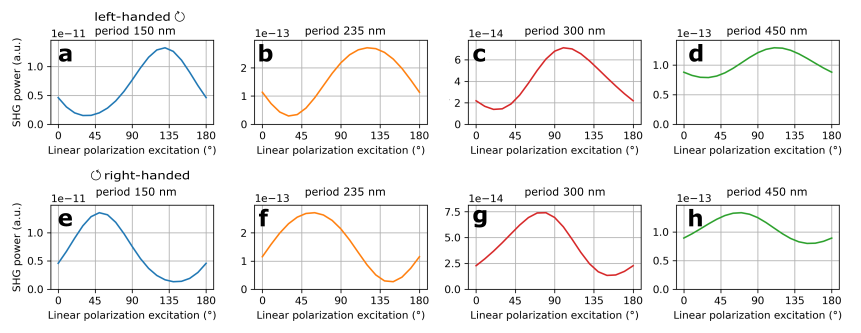

Supplement: Supplementary file 1 — ph4c00721_si_001.zip [file ph4c00721_si_001.zip › Fig_Suppl_PolarPlots.PNG]

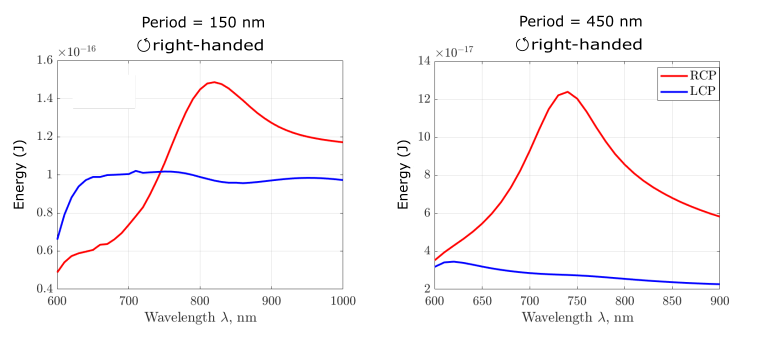

Supplement: Supplementary file 1 — ph4c00721_si_001.zip [file ph4c00721_si_001.zip › Fig_Suppl_Energy.PNG]

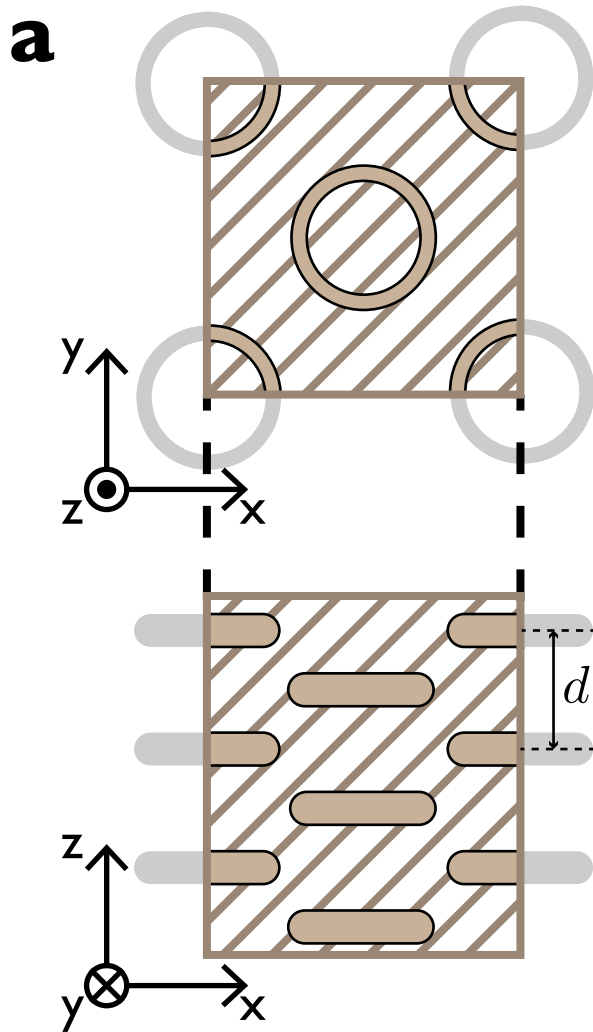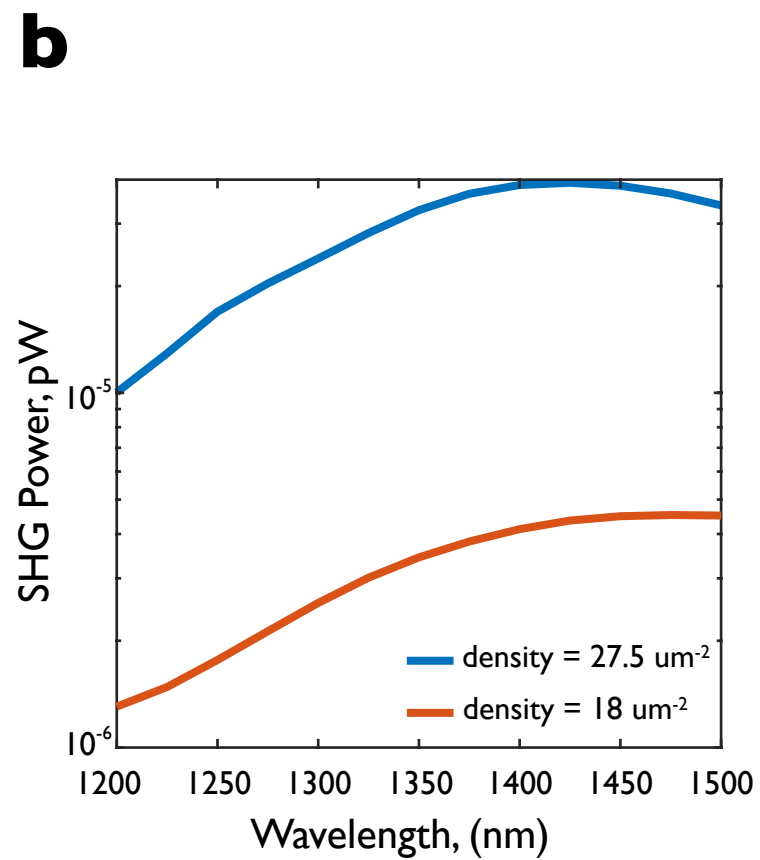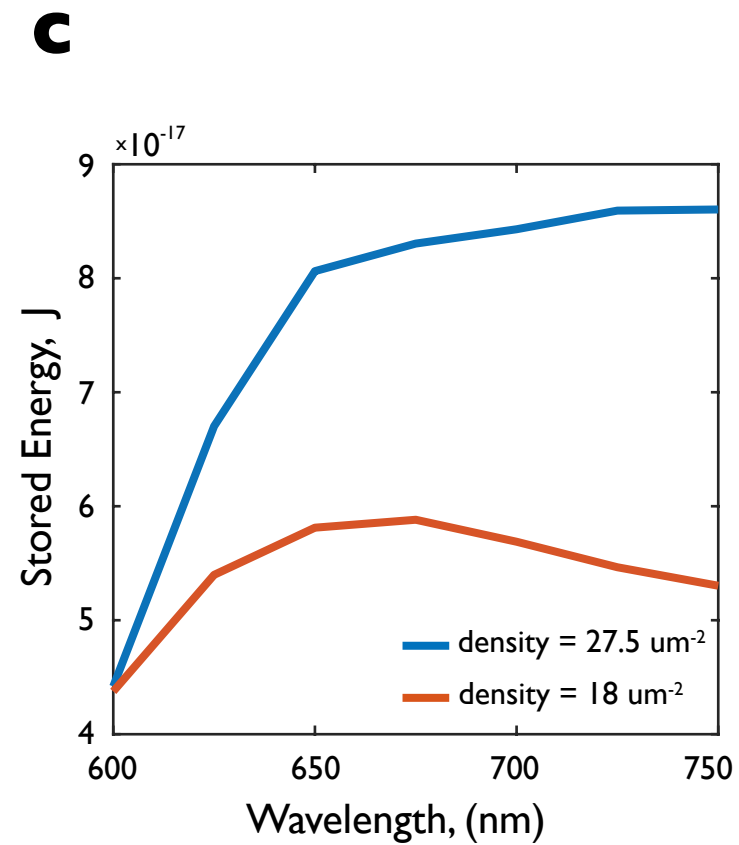

Supplement: Supplementary file 1 — ph4c00721_si_001.zip [file ph4c00721_si_001.zip › tori_numerical.pdf]

# Simulation

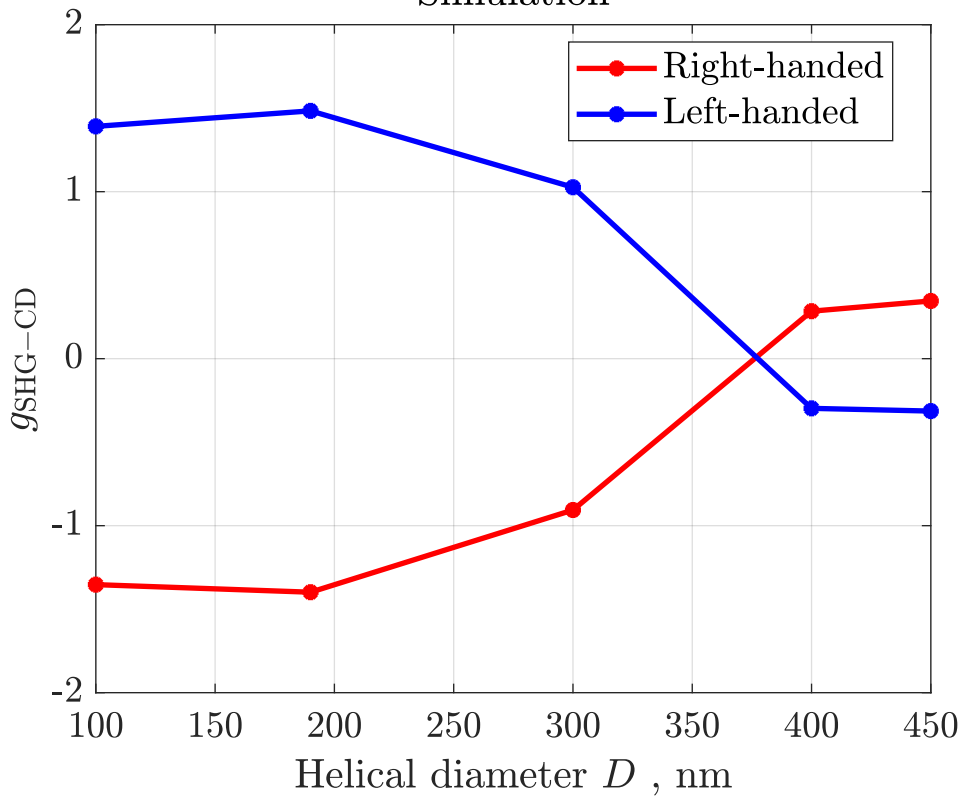

Supplement: Supplementary file 1 — ph4c00721_si_001.zip [file ph4c00721_si_001.zip › Fig_Suppl_fix_spacing.pdf]

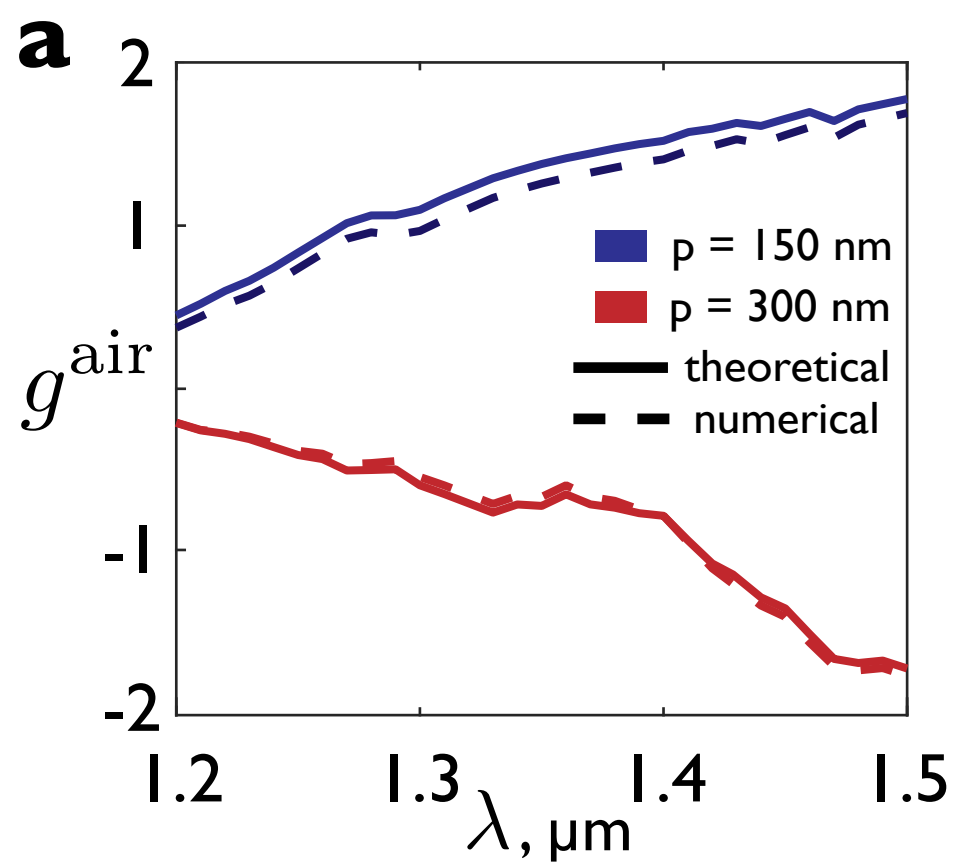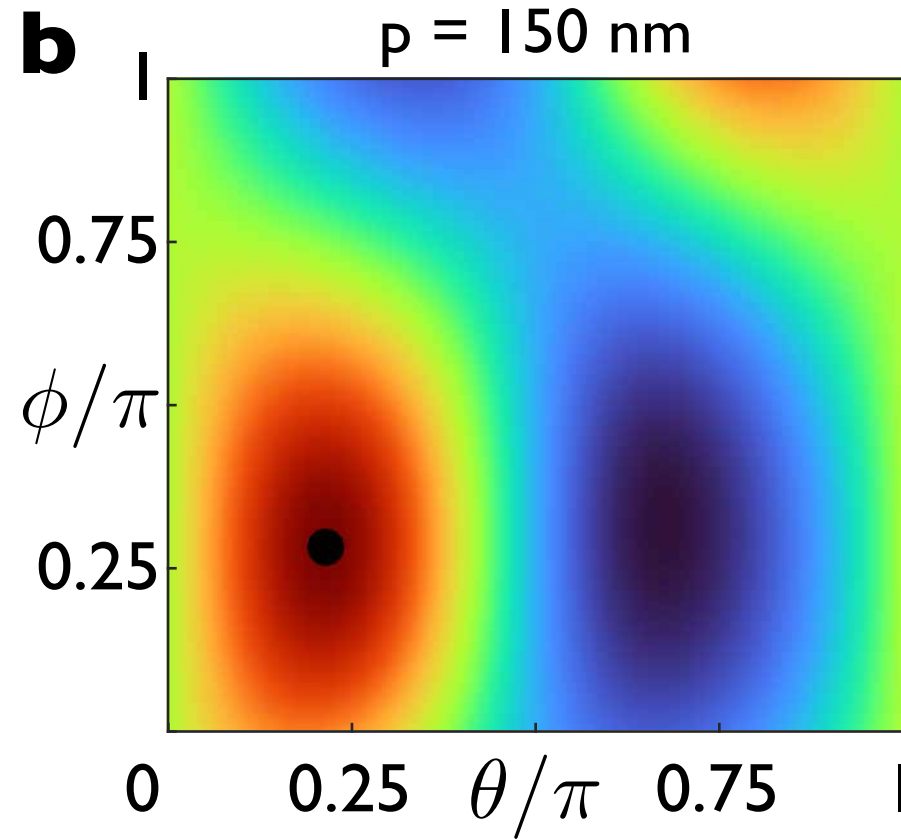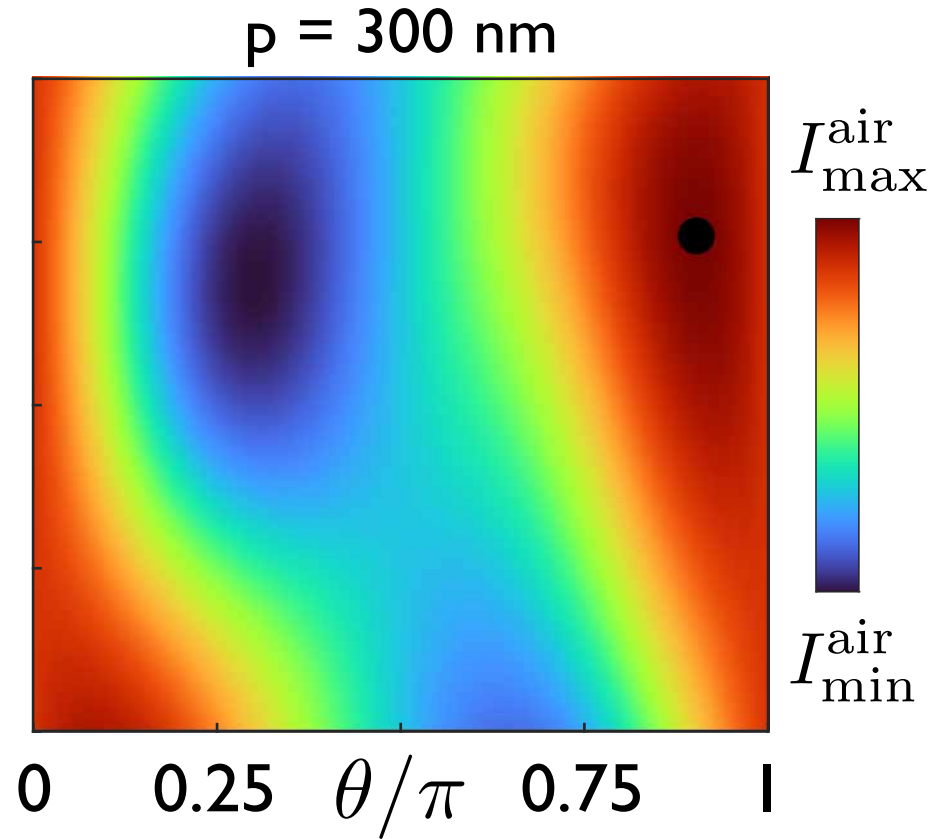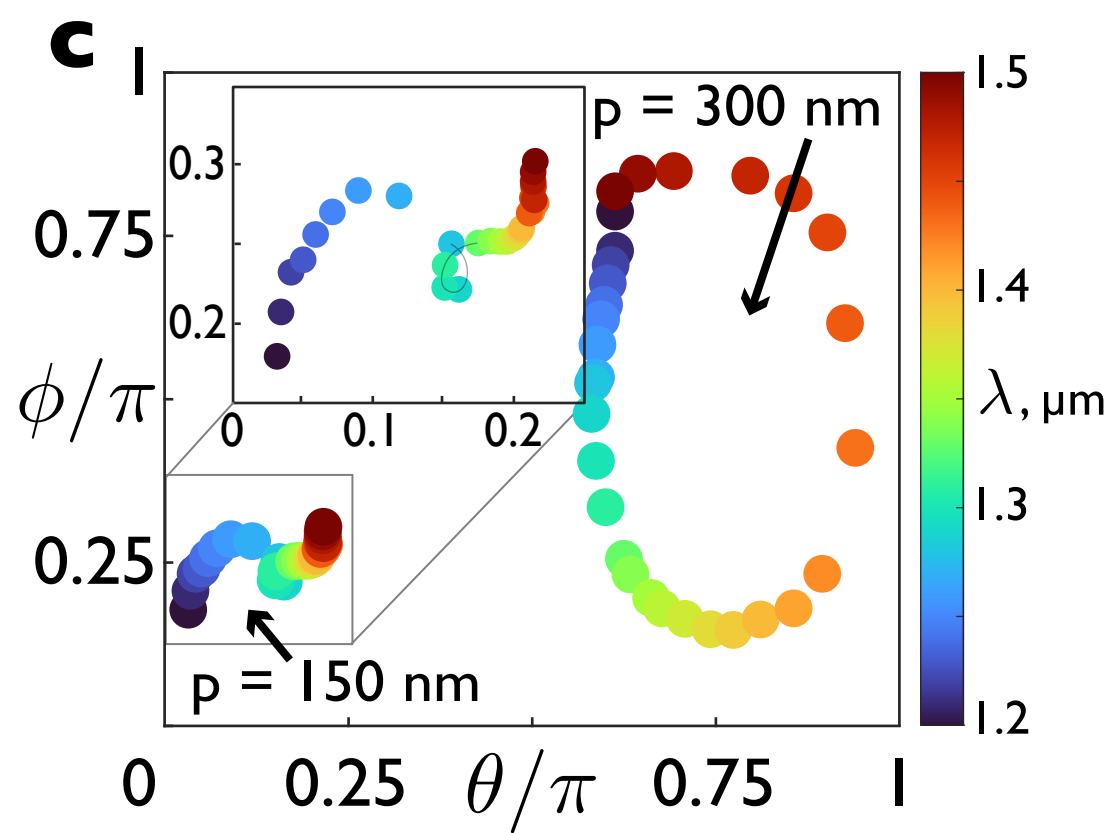

Supplement: Supplementary file 1 — ph4c00721_si_001.zip [file ph4c00721_si_001.zip › Fig_Suppl_IG.pdf]

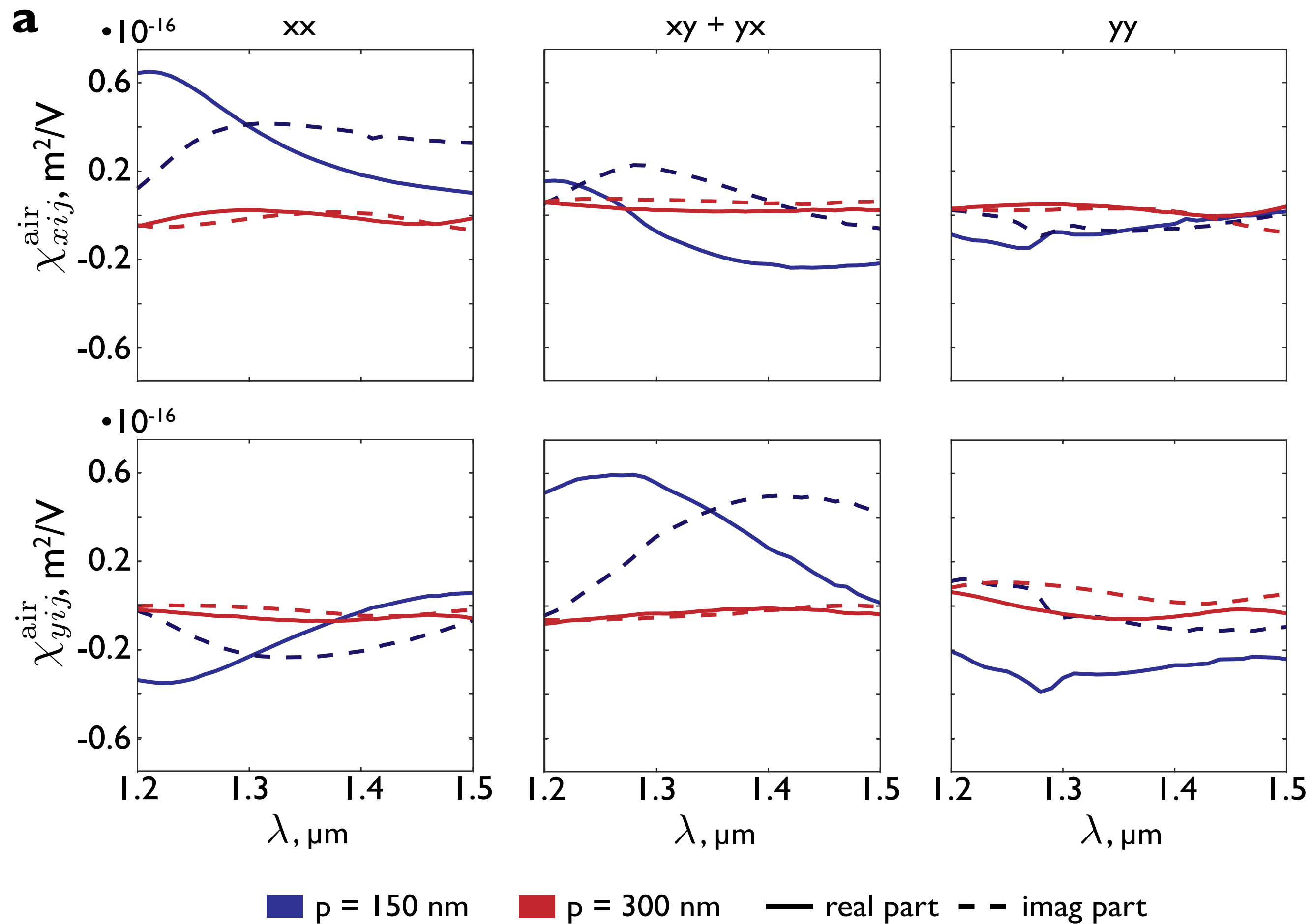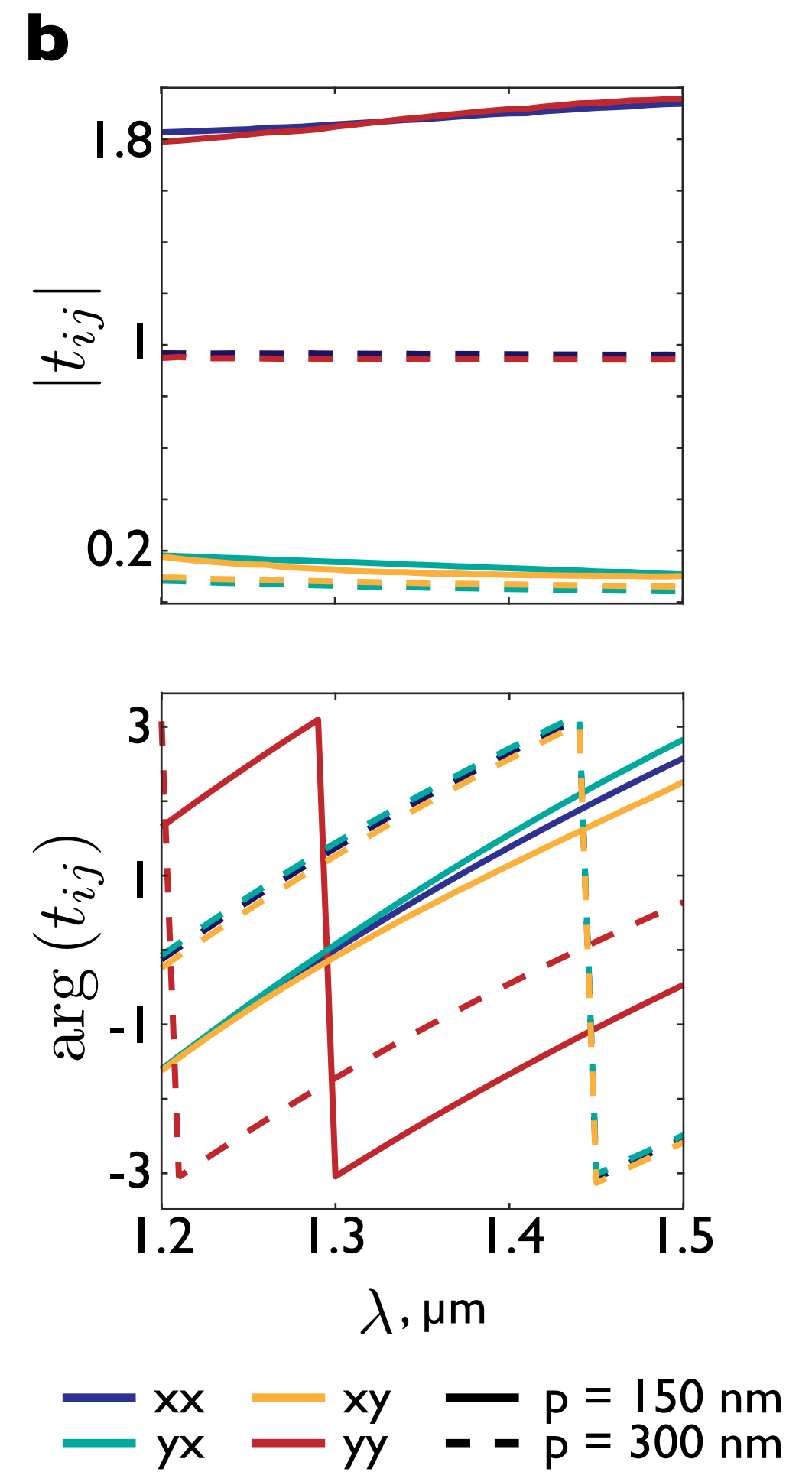

Supplement: Supplementary file 1 — ph4c00721_si_001.zip [file ph4c00721_si_001.zip › Fig_Suppl_EffectiveChi2.pdf]
